# Women Inspectors under the Factory Acts

**Published:** 1898-07-30

**Authors:** 


### WOMEN INSPECTORS UNDER THE FACTORY ACTS.

In answer to a question regarding the amount of work done by the women inspectors under the Home Office in 1897, and the number of instances in which applications had been made to them by women workers, Sir M. W. Ridley said that of the complaints made 400 related to matters within the scope of the Factory and allied Acts, and of these 400, 159 came direct from the workpeople or their friends, 122 from various industrial organisations, 20 from public officials, and 99 were anonymous. It is satisfactory to note that a considerable proportion of the complaints came direct from the workpeople, for it must be recognised that one great reason for the appointment of women inspectors is that it is hoped that they will gain the confidence of the women workers, and thus become more fully cognisant of all that goes on within the factories than is possible in the case of male inspectors, who can but act upon what they see. Factory and Workshops Acts must always be more or less ineffectual unless the workpeople themselves take an interest, and even a part, in their administration.

